# Folie à Deux in the Family Environment: A Case of Shared Delusion Between a Mother and Her Son

**DOI:** 10.7759/cureus.78703

**Published:** 2025-02-07

**Authors:** Christian Galindo, Lesmer Galindo

**Affiliations:** 1 Psychiatry, Corporación Universitaria Remington, Medellin, COL; 2 General Practice, Corporación Universitaria Remington, Medellin, COL

**Keywords:** folie à deux, pathological family dynamics, schizoaffective disorder, shared psychotic disorder, substance use disorder (sud)

## Abstract

Shared psychotic disorder (SPD), also called folie à deux, is an uncommon clinical entity characterized by the transmission of delusions from one psychotic individual to another within the framework of a close relationship, typically isolated from the social environment. Although rare, this phenomenon poses a significant diagnostic and therapeutic challenge, particularly in family settings. This report presents a clinical case of folie à deux between a mother and her child, highlighting the complexities of therapeutic management and providing a succinct review of the literature to adequately frame the clinical approach. The report illustrates the need for a comprehensive therapeutic intervention involving both the management of the underlying psychotic disorder and the dissolution of pathological family dynamics that perpetuate shared delusions. Additionally, the importance of a multidisciplinary approach is emphasized, as shared delusions can involve multiple members of a nuclear family, increasing the risk of negative consequences at both individual and social levels.

## Introduction

Shared psychotic disorder (SPD), also known as folie à deux, is a rare psychiatric condition in which two or more individuals share the same delusion. Typically, one person - usually the dominant one - has a primary psychotic disorder, while the others adopt their delusions. This unusual and complex condition has captured the attention of medical professionals for centuries. Its earliest records date back to the 16th century, with descriptions by William Harvey [1]. Later, in the 19th century, Baillarger formally identified the disorder under the term folie communiquée. However, in 1877, the French psychiatrists Lasègue and Falret coined the term folie à deux [2], which has endured as the most recognized term for this rare clinical condition. This disorder is characterized by the transmission of delusions from a psychotic individual (primary case) to one or more non-psychotic individuals (secondary cases) in the context of a close, prolonged, and generally isolated relationship with the social environment.

In 1942, Alexander Gralnick proposed a classification that has allowed a better understanding of the various forms in which SPD can manifest itself. He categorized SPD into four main subtypes: folie imposée, where the delusions of the psychotic individual are imposed on a non-psychotic individual; folie simultanée, in which two predisposed individuals simultaneously develop an identical psychosis [2]; folie communiquée, which describes the transfer of delusions to another person after a prolonged period of resistance; and folie induite, in which the secondary subject develops new delusions induced by the relationship with the psychotic subject. These subtypes highlight the complexity of SPD, where the pathological relationship between individuals plays a crucial role in the maintenance and evolution of the shared delusion.

Psychiatric literature has used various names to describe SPD, such as “communicated insanity,” “association psychosis,” and “symbiotic psychosis.” Modern classification, according to the Diagnostic and Statistical Manual of Mental Disorders, fourth edition, text revision (DSM-IV-TR) and the International Classification of Diseases tenth revision (ICD-10), includes SPD [3] under codes 297.3 and F24, respectively, categorizing it as induced delusional and induced psychotic disorders. Although SPD is considered a rare disorder, it is likely underdiagnosed [4] due to the reluctance of patients to seek medical help, which is influenced by the lack of introspection and social isolation that often characterizes affected dyads or groups.

This case report aims to contribute to the clinical understanding of SPD by presenting an illustrative case of folie à deux, specifically the subtype folie induite; the interpersonal dynamics and psychopathological factors involved are analyzed. This case highlights the importance of early clinical intervention and the need for a comprehensive therapeutic approach that includes separation of the affected individuals and simultaneous treatment of their underlying conditions.

## Case presentation

A 33-year-old male patient, a native of Medellín and resident of Santa Rosa de Osos, Antioquia, presented with a significant psychiatric history, with diagnoses of schizoaffective disorder and psychoactive substance use disorder, specifically cannabis and tobacco. The patient, with incomplete primary education and sporadic employment in agriculture and mining, was single and childless and lived with his father, who was also involved in mining and agriculture. Occasionally, the patient visited his 57-year-old mother, who resided in Bello, Antioquia.

Since 2008, the patient had undergone multiple hospitalizations, including electroconvulsive therapy (ECTAR) between 2016 and 2017. The patient had so far undergone 24 sessions of bilateral ECT due to exacerbations of his psychiatric disorder, aggravated by active use of cannabis (five cigarettes a day) and tobacco (one pack a day); however, no therapeutic response had been achieved, with no associated side effects reported during the treatment.

**Figure 1:**
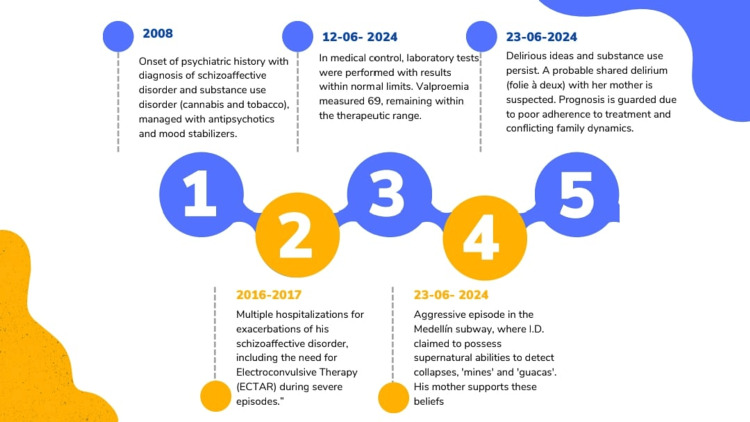
Timeline of the case with shared psychotic features This timeline presents key events in a patient's psychiatric history, including hospitalizations, electroconvulsive therapy (ECT), persistent delusions, substance use, and shared psychotic symptoms with his mother. It highlights episodes of aggression, laboratory findings, and challenges in treatment adherence

Throughout his clinical history, the patient had manifested persistent ideas of harm and persecution, especially toward his father, with episodes of physical aggression. These behaviors had been frequently minimized by his mother, who justified them, stating that “the father stole his potty.” In addition, she claimed that her son possessed supernatural “gifts” to predict events, such as what happened on June 23, 2025, when the patient, in an episode of aggression in the Medellín subway, stated that he “knew” that the cabin would collapse, interpreting it as a manifestation of his supposed ability to detect minerals and mines. His mother also claimed that the patient could identify “mines and guacas”, having conducted exploration on a neighbor's property that had ended in conflict. An illustrated timeline of the case is presented in Figure 1.

The patient's family history included a background of psychiatric disorders. The mother had been diagnosed with schizophrenia 20 years ago; however, she had never attended medical follow-ups. Neither parent had a history of psychoactive substance use. He had been treated with a combination of antipsychotics and mood stabilizers, including clozapine, risperidone, and divalproex sodium. However, his treatment adherence had been inconsistent due to side effects such as drowsiness and his resistance to treatment, which had contributed to the persistence of psychotic symptoms.

During a recent outpatient physical evaluation, the patient was alert, oriented, and with good personal presentation. He exhibited a cooperative attitude, and no sensory-perceptual disturbances were noted at that time. Delusions of harm and persecution persisted, especially in relation to his father, as well as bizarre ideas related to rocks and minerals. The patient denied suicidal or self-injurious ideation during the evaluation. The most recent laboratory tests, conducted on June 12, 2025, revealed values within normal parameters, including a complete blood count (CBC), serum creatinine, blood glucose, and liver transaminase. Additionally, valproic acid levels, assessed on May 27, 2025, showed a valproemia of 69 µg/mL (within the therapeutic range). No other relevant diagnostic findings were reported.

The patient’s diagnosis included schizoaffective disorder, substance use disorder (cannabis and tobacco), and probable shared psychotic disorder (folie à deux), specifically the folie induite subtype, with his mother. This diagnosis presented significant challenges, given that both share similar psychotic symptoms, complicating the differentiation of the sources of delusion. Limited adherence to treatment and active substance use further complicated the management of the clinical picture.

The prognosis is guarded due to the chronicity of symptoms, poor adherence to treatment, and the negative influence of his relationship with his mother. The patient has been managed with clozapine (100 mg nightly), risperidone (2 mg morning and evening), and divalproex sodium (500 mg every 12 hours), with adjustments to risperidone to address persistent symptoms. Despite continued antipsychotic treatment, the patient shows persistence of delusional symptoms, albeit with less structuring after risperidone adjustment. His mother has reported a partial improvement in his behavior, but ideas of harm and referentiality persist. The patient currently denies suicidal ideation or self-harm, and no serious adverse events associated with treatment have been reported. However, nonadherence and resistance to medical intervention significantly complicate the management and prognosis of the case.

This case highlights the complexity of managing folie à deux, especially in the context of poor adherence to treatment and active substance use, which exacerbates psychotic symptoms and hinders long-term recovery. Close psychiatric follow-up and addressing both the patient’s primary disorder and the pathological influence of his relationship with his mother is crucial to prevent future decompensation.

## Discussion

From a psychodynamic and social point of view, SPD poses a significant challenge [5]. The transmission of delirium requires not only a close and prolonged relationship [6] but also a context of social isolation that limits the affected individuals' access to external reality. Generally, the primary case involves a person with a chronic psychotic illness, such as paranoid schizophrenia or a delusional disorder, and usually presents a dominant personality that facilitates the transmission of delirium. The secondary individual, on the other hand, tends to be more suggestible, with lower self-esteem and a more passive personality, which predisposes them to accept the delusional beliefs of the primary case.

SPD is not exclusively limited to dyads; there are documented cases of folie à trois, folie à quatre, and folie à famille [7], which underline the versatility and depth of this clinical condition. These variants are more common in nuclear families, especially between spouses, mothers and children, or siblings, suggesting the influence of genetic and psychosocial factors in its etiology. Epidemiologically, the literature is scarce and mainly based on case reports, which limits the ability to obtain accurate data on its prevalence and incidence. However, existing studies suggest that SPD is more common than believed, although its diagnosis remains a challenge due to the lack of specific diagnostic tools and the complexity of the underlying mechanisms.

The treatment of SPD [8] should be comprehensive and multifaceted, focusing on treating the primary illness of the delusional patient and dismantling the pathological relationship that perpetuates the shared delusion. Physical separation of affected individuals, although necessary, must be carefully managed to avoid psychological trauma. In addition, treatment should include both psychopharmacology and psychotherapy, with a focus on improving the autonomy and critical capacity of the secondary patient.

This case provides a detailed insight into the complexity of SPD, also known as folie à deux, highlighting both the strengths and limitations of clinical management in such situations [9,10]. One of the key strengths of this report lies in the identification of a pathological dynamic within the nuclear family that perpetuates the shared delusions between the patient and the mother. This identification has allowed for more targeted clinical intervention, which is crucial for addressing such an intricate disorder effectively [11]. However, the management of this case also presents significant challenges. One of the primary difficulties has been the patient's inconsistent adherence to treatment, a common obstacle in individuals with chronic psychotic disorders, especially when coexisting with active substance use. Poor adherence complicates clinical management and limits the effectiveness of therapeutic interventions, as evidenced by the persistence of delusional symptoms despite pharmacological adjustments [12].

The medical literature highlights that SPD is a rare but underdiagnosed condition, primarily due to the intricate dynamics of relationships that sustain it and the difficulty in distinguishing between the sources of shared delusions [13]. In this context, this case underscores the importance of early and accurate diagnosis, which must be supported by a thorough understanding of family dynamics. Diagnosis in this case was further complicated by the symmetry of psychotic symptoms between the patient and the mother, making it difficult to differentiate between primary and secondary sources of delusions. This diagnostic challenge is well-documented in the literature, indicating that social isolation and the dominant influence of the primary case are key therapeutic targets for breaking the cycle of shared delusions.

From a scientific perspective, the conclusions drawn from this case are supported by the persistence of psychotic symptoms despite medical intervention and the pathological relationship between mother and patient [14]. The assessment suggests that family dynamics, marked by the mother's belief in the patient's “gifts,” play a central role in perpetuating shared delusions. Additionally, the active use of cannabis and tobacco exacerbates symptoms and reduces treatment effectiveness, highlighting the need for a comprehensive approach that addresses both psychotic disorder management and substance abuse rehabilitation.

This case offers important lessons for clinical practice. Firstly, early identification and proper management of family dynamics in cases of SPD are essential to prevent the perpetuation of shared delusions. Second, treatment adherence is crucial; clinicians must work closely with the patient and their family to ensure that therapeutic guidelines are followed and to mitigate the influence of external factors, such as substance use. Finally, this case underscores the importance of a multidisciplinary and continuous approach, integrating both pharmacological and psychotherapeutic interventions, with a special focus on dismantling the pathological relationship that sustains the shared delusion.

## Conclusions

This case underscores the complexity of managing SPD (folie à deux), particularly in the setting of comorbid schizoaffective disorder and psychoactive substance use (cannabis and tobacco). The pathological dyadic interaction with the mother, characterized by mutual reinforcement of delusions, plays a key role in symptom persistence and treatment resistance. While substance use and potential environmental factors may have contributed to the clinical picture, the predominant findings indicate SPD as the primary diagnosis. This aligns with existing literature on the impact of shared psychotic phenomena in family dynamics, reinforcing the importance of distinguishing primary psychotic disorders from those induced by interpersonal relationships.

The management requires a multidisciplinary approach, integrating pharmacological stabilization, targeted psychosocial interventions, and structured efforts to modify dysfunctional family dynamics. The timing and effectiveness of early interventions are discussed in this report, highlighting how delayed recognition of folie à deux can contribute to chronicity and poor prognosis. To improve long-term outcomes, sustained therapeutic adherence and continuous psychiatric monitoring remain essential. Additionally, this report contributes to broader clinical knowledge by demonstrating the challenges of differential diagnosis in SPD and the need for individualized treatment strategies, particularly in patients with coexisting substance use.
